# Effect of Applying Struvite and Organic N as Recovered Fertilizers on the Rhizosphere Dynamics and Cultivation of Lupine (*Lupinus angustifolius*)

**DOI:** 10.3389/fpls.2020.572741

**Published:** 2020-11-19

**Authors:** Ana A. Robles-Aguilar, Oliver Grunert, Emma Hernandez-Sanabria, Mohamed Mysara, Erik Meers, Nico Boon, Nicolai D. Jablonowski

**Affiliations:** ^1^Department of Green Chemistry and Technology, Faculty of Bioscience Engineering, Ghent University, Ghent, Belgium; ^2^Forschungszentrum Jülich GmbH, Institute of Bio- and Geosciences, IBG-2: Plant Sciences, Jülich, Germany; ^3^Center for Microbial Ecology and Technology, Ghent University, Ghent, Belgium; ^4^Greenyard Horticulture, Ghent, Belgium; ^5^Laboratory of Molecular Bacteriology, VIB – KU Leuven Center for Microbiology, Rega Institute, Leuven, Belgium; ^6^Unit of Microbiology, Belgian Nuclear Research Center, StudieCentrum voor Kernenergie⋅Centre d’étude de l’Energie Nucléaire (SCK⋅CEN), Mol, Belgium; ^7^Department of Bioscience Engineering, Vrije Universiteit Brussel, Brussels, Belgium

**Keywords:** lupine, growing medium, soilless culture systems, fertilizer, microbial communities, nitrogen

## Abstract

Intensive agriculture and horticulture heavily rely on the input of fertilizers to sustain food (and feed) production. However, high carbon footprint and pollution are associated with the mining processes of P and K, and the artificial nitrogen fixation for the production of synthetic fertilizers. Organic fertilizers or recovered nutrients from different waste sources can be used to reduce the environmental impact of fertilizers. We tested two recovered nutrients with slow-release patterns as promising alternatives for synthetic fertilizers: struvite and a commercially available organic fertilizer. Using these fertilizers as a nitrogen source, we conducted a rhizotron experiment to test their effect on plant performance and nutrient recovery in lupine plants. Plant performance was not affected by the fertilizer applied; however, N recovery was higher from the organic fertilizer than from struvite. As root architecture is fundamental for plant productivity, variations in root structure and length as a result of soil nutrient availability driven by plant–bacteria interactions were compared showing also no differences between fertilizers. However, fertilized plants were considerably different in the root length and morphology compared with the no fertilized plants. Since the microbial community influences plant nitrogen availability, we characterized the root-associated microbial community structure and functionality. Analyses revealed that the fertilizer applied had a significant impact on the associations and functionality of the bacteria inhabiting the growing medium used. The type of fertilizer significantly influenced the interindividual dissimilarities in the most abundant genera between treatments. This means that different plant species have a distinct effect on modulating the associated microbial community, but in the case of lupine, the fertilizer had a bigger effect than the plant itself. These novel insights on interactions between recovered fertilizers, plant, and associated microbes can contribute to developing sustainable crop production systems.

## Introduction

Intensive agriculture and horticulture heavily rely on the input of fertilizers to sustain food (and feed) production (Vaneeckhaute et al., 2013). The global demand for fertilizers amounts to an estimated 110 million tons (Mt) of N, 47.0 Mt P_2_O_5_, and 37.5 Mt K_2_O per year (Spanoghe et al., 2020). However, high carbon footprint and pollution are associated with the mining processes of P and K, and the artificial nitrogen fixation for the production of synthetic fertilizers (Pikaar et al., 2018). Organic fertilizers or recovered nutrients from different waste sources can be used to reduce the environmental impact of fertilizers (Burnett et al., 2016; Tittarelli et al., 2016). Also, these nutrient recovery techniques can yield high-performance, flexible, and concentrated mineral fertilizers such as struvite (MAP, NH_4_MgPO_4_⋅6H_2_O), with a demonstrated high fertilizer use efficiency, also considering the plants’ nutrient release and uptake strategies (Sigurnjak et al., 2016; Vaneeckhaute et al., 2016; Robles-Aguilar et al., 2019a).

Commercial inorganic fertilizers are high in nutrient content, easily soluble, rapidly available, and have low and competitive prices, rendering them in principle more effective and efficient than organic or recycled fertilizers. However, organic fertilizers release nutrients slowly (Dion et al., 2020), lowering P fixation and N losses via leaching. Moreover, they enhance root growth and improve soil structure and water holding capacity, reducing soil acidification (Chen, 2006; Paungfoo-Lonhienne et al., 2012). Similarly, many studies have demonstrated struvite (recycled fertilizer) as a crystalline mineral free of contaminants that can be used as a slow-releasing fertilizer (El Diwani et al., 2007; Robles-Aguilar et al., 2019b).

Nitrogen is generally considered the main factor limiting plant growth. Struvite, generally seen merely as a P fertilizer, also contains NH_4_^+^ (6.5%). The N cycle (dominated mainly by microbial processes) has a significant impact on soil chemistry and, consequently, on soil fertility (Jetten, 2008). Therefore, analyzing the N dynamics after struvite application is crucial in order to define the overall fertilizer efficiency.

Organic fertilizers, next to synthetic fertilizers, are an important supplier to the modern horticultural industry. The combination of organic fertilizers in growing media is not always easy, as the delivery of nutrients depends on microbial breakdown and interaction. Similarly, in order to assure optimal plant performance, the effect of incorporating recovered fertilizers such as struvite in the growing media needs to be analyzed taking into account its influence in the bacterial community (Van Gerrewey et al., 2020).

Narrow-leafed lupine (*L. angustifolius* L.) is a native European legume, with a high seed protein content (up to 44%) (Lucas et al., 2015). Plant proteins have been presented as a sustainable alternative to animal protein; however, Europe still depends on the import for 70% of its plant protein requirements (mainly import of soybean). Therefore, the growth of lupine contributes to the sustainability of cropping systems (Lucas et al., 2015), representing an effective alternative to other crops like soy. In our previous studies, we could demonstrate that *L. angustifolius*, by exudation of organic acids, allowed for an improved P release and uptake from struvite (Robles-Aguilar et al., 2019a). The combination of its high protein quality, its ability to mobilize nutrients, and the capacity to take up nutrients from recycled fertilizers makes lupine a particularly promising crop to meet sustainability and circular economy goals.

Lupine is known for its high physiological root plasticity, related to exudation of large amounts of organic acids that, for example, can free P from insoluble forms (Lambers et al., 2012; Robles-Aguilar, 2018). However, lupine root systems can influence not only the nutrient turnover but also the rhizosphere microbial composition and pH. The significance of the rhizosphere (Hiltner, 1904) arises from the volume of soil influenced by exudates from plant root tissues and colonized by rhizobacteria and subsequently altering microbial activity, nutrient cycling, and plant growth. Consequently, the concentration of nutrients with a cycle highly affected by the microbial activity like N may differ between rhizosphere and the bulk growing medium. Particularly the growing medium directly attached to the roots named the rhizosheath is dynamically influenced by plant–microbiome interactions as observed in a previous investigation on tomato (Grunert et al., 2019). In that study, it was shown that the presence of a tomato plant leads to a convergence toward a similar microbial community, regardless of fertilization (Grunert et al., 2019), indicating that the plant rather than the fertilizer shifted the microbial community in the growing medium. However, the microbial composition can also be affected by the type of growing media used (Grunert et al., 2016b) or by the cultivation practice, meaning the type of fertilization, as shown by Edwards et al. (2015) in field conditions.

Plant growth performance counts substantially on the availability of nutrients at the soil–root interface, which in turn is shaped by a wide range of factors including the soil or growing medium characteristics, environmental conditions, and the microbial community and its structure (Grunert et al., 2016a). The function of the microbial community as fertilizer can be direct and indirect (Sakarika et al., 2019). The first is associated with the use of dead biomass, with its inherent nitrogen/phosphorus/potassium content. Dead microbial biomass having a direct fertilizer function can be used by plants as nitrogen/phosphorus/potassium (N/P/K) source. Living biomass can also boost the nutrient acquisition, due to microbial activities such as nitrogen fixation and P solubilization. Biological N_2_ fixation can occur primarily in soil by either free-living or plant-associated diazotrophs (Galloway et al., 2008). Symbiotic bacteria (e.g., Rhizobia) fix nitrogen inside nodules and are hence endosymbionts. Non-symbiotic nitrogen fixing microbes are generally referred as diazotrophs and are identified as free-living within soil or associated with plant roots (Vadakattu et al., 2019) and include Cyanobacteria, Proteobacteria, Archaea, and Firmicutes (Pii et al., 2015).

Plants can use a wide array of chemical N forms, ranging from simple inorganic N compounds such as ammonium (NH_4_^+^), nitrate (NO_3_^–^) to polymeric N forms such as proteins (Paungfoo-Lonhienne et al., 2008, 2012). The primary nitrogen forms taken up by the plant are ammonium and nitrate (Marschner, 2011). As in soils with a large organic N pool and growing media supplemented with organic fertilizers, it is indisputable that the organic nitrogen is only made accessible to the plants by the decomposition carried out by bacteria with first the ammonium as a side product and subsequent nitrification. Microorganisms like mycorrhizal fungi and plant growth-promoting rhizobacteria mineralize organic nitrogen by releasing hydrolytic enzymes and thus enhancing the nutrient availability in soil (Miransari, 2011; Ollivier et al., 2011) and most likely also in other culture systems such as soilless growing medium.

This study aims to elucidate the effect of organic fertilizer, and a mineral fertilizer recovered from wastewater, i.e., struvite, on the below and aboveground plant development of lupine, its root architecture, and nutrient turnover. Furthermore, our current understanding of the factors affecting microbial communities inhabiting growing media and how, in turn, it influences plant nitrogen availability, is still limited. Hence, this study also aims to determine the effect of an organic fertilizer and an inorganic recovered fertilizer on the root-associated microbial community structure and its functionality.

We hypothesized that (a) the cultivation of lupine will be affected differently by both fertilizer sources of nitrogen (organic vs. inorganic) due to different N release dynamics in the rhizosphere. The organic fertilizers have in common that the major part of the N and P is bounded in complex molecules such as proteins; consequently, their release is related to the decomposition by the microbial community associated with the soil. Therefore, we hypothesized that (b) the microbial community in the rhizosphere (volume of growing medium influenced by the root)/rhizosheath (growing medium directly attached to the roots) will have a more significant influence on the nutrient turnover of the organic fertilizer compared with the struvite. Finally, we hypothesized that (c) lupine plants will influence the microbial community structure in the growing medium compared with the medium with no plants.

## Materials and Methods

For this experiment, we applied the methods described in Grunert et al. (2019), as experiments were conducted under the same conditions.

### Fertilizer Treatments and Used Growing Medium

The struvite used in this study was recovered from municipal wastewater, following anaerobic digestion, solid–liquid separation, and nitrogen removal, provided by The Laboratory of Chemical and Environmental Engineering, Lequia, University of Girona, Spain. The commercially available organic fertilizer (Frayssinet, France) is based on vegetable and animal-derived material, sugar beet vinasse, press cake, fruit pulp, and composted manure. The chemical composition of the recovered nutrients contained in the fertilizers used is detailed in Table 1.

**TABLE 1:** Chemical composition of the recovered nutrients.

Parameters (% FW)	Organic fertilizer (ORG) (mean ± stdev)	Struvite (NH_4_MgPO_4_⋅6H_2_O) (STR) (mean ± stdev)
Total N (%)	7.780.19	ND
Organic-N (%)	6.890.17	ND
NH_4_-N (%)	0.360.02	6.60.2
NO_3_-N (%)	0.0170.001	ND
Urea-N (%)	0.510.03	ND
P in mineral acid (%)	2.190.06	13.20.4
K in water (%)	4.930.12	ND
Ca total (%)	5.700.14	ND
Mg total (%)	0.570.03	10.60.2
S total (%)	2.140.05	ND
Na total (%)	0.480.02	ND
Organic matter (%)	54.41.4	ND

**ND, not determined; stdev, standard deviation, n = 3. Values refer to weight-%.**

The organic growing medium (GB, Grow Bag, Greenyard, Belgium) consisted of a mixture of peat (H2–H4 on the von Post scale70 [40% v/v], Irish peat [40% v/v], and coconut fiber [20% v/v]). The average fresh bulk density (*n* = 4) of the growing medium was 225.04 kg/m^3^, determined according to EN12580. The growing medium had a gravimetric water content of 0.50 ± 0.02 kg kg^–1^. Fertilizers were mixed with the organic growing medium at a dose of 100 mg N L^–1^ growing medium (equivalent to 300 kg N ha^–1^ in arable soil or 0.7 kg/m^3^ compound fertilizer (PGMIX 14-16-18, Yara, Norway). The amount of 100 mg N L^–1^ of growing medium is common practice in the growing media industry and is used for base fertilization of growing media (Sonneveld and Voogt, 2009). The organic growing medium was mixed with the lime [(Ca, Mg)CO_3_)_2_] with a portable concrete mixer to adapt the pH(H_2_O) to 5.6. The chemical composition of the growing medium is described in Table 2.

**TABLE 2:** Chemical analyses of the organic growing medium (GB, Grow Bag, Greenyard, Belgium) after fertilizer addition.

Parameters	GB without fertilizer (NoF)	GB with organic fertilizer (ORG)	GB with struvite (STR)
pH (H_2_O)	5.5	5.7	5.6
Electrical conductivity (μS cm^–1^)	113	115	186
NO_3_-N (mg N L^–1^)	0	0	0
NH_4_-N (mg N L^–1^)	1.71	18.4	42.1
P (mg P L^–1^)	18.7	27.3	203.1
K (mg K L^–1^)	107.5	195	115
Ca (mg Ca L^–1^)	835	747.5	607.5
Mg (mg Mg L^–1^)	235	242.5	332.5
SO_4_ (mg SO_4_ L^–1^)	140.5	196	126.9
Na (mg Na L^–1^)	52.5	67.5	57.5
Cl (mg Cl L^–1^)	105.9	102.8	104.2

**Lime [(Ca, Mg)CO_3_)_2_] was added at a dose of 2.5 kg m^−3^ and mixed with a portable concrete mixer to adapt the pH(H_2_O) to 5.6.**

All the other necessary nutrients were added as Hoagland nutrient solution. The formulation of the solution was calculated according to an N:P:K ratio of 1:0.5:1.1. Gamma irradiation (BGS, Wiehl, Germany) at a minimal dose of 50 kGy was used to eliminate the native microbial community in the growing medium of the control treatment (sterile). Subsequently, each rhizotron was filled with 1.3 kg of the prepared growing medium (equaling 5 L per rhizotron).

For each treatment (i.e., struvite, organic, and no fertilizer), 10 rhizotrons were prepared. An equal number of rhizotrons were filled and placed as described above but without any plants. These served as unplanted controls to follow the soil bacterial community and nutrient turnover dynamics, making a total of 60 Rhizotrons (30 with plants and 30 without plants).

Lupine plants (*Lupinus angustifolius* L. subsp. *angustifolius*, cultivar: blau “Boregine,” Kiepenkerl, Germany) were grown in rhizotrons employing the same conditions as described in Grunert et al. (2019). Lupine seeds were planted at 2 cm depth and in contact with the Plexiglas. Rhizotrons were maintained at an angle of 45° during the whole growing period to ensure the maximum number of visible roots growing along the transparent surface. All plants were supplied with water, and each rhizotron received 100 mL of 1/3 Hoagland nutrient solution at the beginning of the experiment, following 60 mL three times per week to keep a soil volumetric water content of ∼30% (VWC).

Time point 0 was considered as the time when the rhizotrons were filled, and the seeds were placed into the rhizotron (i.e., the start of the experiment); 14 days after sowing (DAS), 50% of all the rhizotrons (i.e., 5 rhizotrons per treatment) were opened to collect the microbial and growing medium samples (i.e., time point 1) and the first harvest of lupine plants was performed. Two weeks after the first harvest, i.e., 27 DAS, the remaining 50% of the rhizotrons were harvested (i.e., the second harvest) to collect the plant material and microbial and growing medium samples again. At this point, the experiment was terminated because the plant roots reached the bottom of the rhizotrons.

### Scanning of the Root Architecture

The first roots were visible at the transparent surface of the rhizotrons 7 days after sowing. At that point the measurement of the roots started. In total, the roots were measured at six time points (7, 10, 12, 14, 20, and 27 DAS).

The total root length (sum of seminal and lateral root length) was measured non-invasively by tracing the roots visible at the transparent surface, and the root length was determined by scanning the picture of the Plexiglas and analyzing the visible root length using WinRhizo software (WinRhizo, Regent Instruments Inc., Canada). The visible root length at the surface of the rhizotron represented only a minor part of the plants’ total root system length. The % of visible root length was measured by eye, meaning without mechanical/procedural support. We assume that roots growing on the surface of the rhizotrons were around 30% of the total root length, based on the roots observed once the rhizotrons were opened and the soil was washed out. The estimated percentage is consistent with previous scientific measurements of the visible root length (Hurd, 1964; Nagel et al., 2012).

Visual scoring of nodulation was done at harvest using the field guide for nodulation and nitrogen fixation (BC Ministry of Forests - Research Branch, 1991). Analyses of the ^15^N were done in 3 replicates of each treatment at time point 2 to determine if the nitrogen of the plant tissue originated from the symbiotic microbial atmospheric nitrogen fixation, as described in a previous study employing legumes as green fertilizer (Nabel et al., 2016). If the legume is fixing N_2_, the foliar d^15^N should reflect the atmospheric N source (0‰).

### Plant Performance and Nutrient Turnover

Shoot biomass, as indicative of plant growth, is considered one of the main traits to assess differences in the N uptake between fertilizers. Therefore, we measured shoot biomass at two time points (14 and 27 DAS) and compared this metric between plants supplemented either with ammonium in the form of struvite or indirectly by the organic N fertilizer. Leaf area was also determined at time point 1 and time point 2. Fresh weight was measured directly after harvesting (Mettler Toledo XS205, Gießen, Germany). Subsequently, leaf area was determined using a leaf area meter (Li-3100, Li-cor, Nebraska, United States). Plant samples were dried at 65°C in a forced-draft oven until dry weights were stable.

Chemical analyses of the growing medium at the different conditions were analyzed at time point 0 (after mixing), time point 1 (harvest 1), and time point 2 (harvest 2). Electric conductivity EC (EN 13038) and pH (H_2_O) (EN 13037) were measured in a 1:5 soil-to-water (v/v) suspension. The pH of the growing medium was determined using standard electrodes (Hanna Instruments pH 209 pH meter, Vöhringen, Germany), using 1:5 distilled water extract at 20°C. Extraction (1:5 v/v) of water-soluble nutrients and elements (NO_3_-N, NH_4_-N, Cl, Na, SO_4_, and PO_4_-P) was done according to EN 13652 and measured by ICP-OES (VarioELcube, Elementar, Langenselbold, Germany). Plant available concentrations of P, K, Ca, Mg, Fe, and Mn were extracted (1:5 v/v) in ammonium acetate buffered at pH 4.65. Nitrate was measured with a continuous flow analyzer (Fiastar 5000, Foss, Denmark). Ammonium was measured by steam distillation (Bremner and Keeney, 1965).

Nutrient contents of plant samples were determined by element analysis via inductively coupled plasma optical emission spectrometry (ICP-OES; VarioELcube, Elementar, Langenselbold, Germany).

Chemical analyses of selected mineral nutrient content in leaves and other parts of the plant allow for the calculation of nutrient recovery, i.e., the percentage of applied nutrients taken up by the plant (White and Hammond, 2008), which can also be related to the efficiency of the applied fertilizer (Marschner, 2011). To analyze the N balance, the N recovery was calculated using the following formula:

$$\mathrm{F1}:\frac{N\,u\,p\,t\,a\,k\,e\,a\,t\,f\,e\,r\,t\,i\,l\,i\,z\,e\,d\,p\,l\,a\,n\,t\,s-N\,u\,p\,t\,a\,k\,e\,a\,t\,n\,o\,n\,f\,e\,r\,t\,i\,l\,i\,z\,e\,d\,p\,l\,a\,n\,t\,s}{N\,a\,p\,p\,l\,i\,e\,d}$$

### Bacterial Community Structure Analysis in the Lupine Rhizosphere and Rhizosheath

The growing medium samples were collected as follows: at the bulk zone (i.e., rhizotrons containing only growing medium without plants) and at two distances from the root: (a) “rhizosphere” zone (region at approximately 1 cm distant from the root) and (b) “rhizosheath” zone samples taken directly at the root (less than 1 mm distance to a root).

Samples from the rhizosheath and rhizosphere zone were obtained using tweezers and scalpels sterilized with 70% ethanol. To collect samples from the rhizosheath, roots were first cut with a sterilized scalpel to avoid contamination. To collect only the growing medium directly attached to the root, the whole cut root was directly stored at −80°C. Afterward, the fresh weight of the growing medium adhering to the roots was quantified by separating the growing medium attached. All samples for chemical and microbial analyses were taken in triplicate per zone sampled per rhizotron (*n* = 3). The fresh weight of each sample was determined, and samples were immediately stored at −80°C for microbial community analysis, described as follows. Two paired samples from each of the 30 samples with the plants and 1 sample from each of the 30 samples without the plant were taken in triplicate, resulting in 2 × 3 samples with plants, and 1 × 3 samples without plants, respectively.

Total DNA was extracted from the growing medium samples using the Power Soil DNA Isolation Kit (MoBio Laboratories Inc., Carlsbad, CA, United States), following the manufacturer’s instructions. We used 500 mg from the rhizosphere and 100 mg from the rhizosheath samples. The concentration and quality of DNA were measured based on the absorbance at 260 and 280 nm in a NanoDrop ND 1000 spectrophotometer (NanoDrop Technologies, Wilmington, DE, United States).

High-throughput amplicon sequencing of the V3–V4 hypervariable region and library generation were performed as described in Grunert et al. (2019). Bioinformatics and data preprocessing followed a protocol developed in-house (Mysara et al., 2017; El Hage et al., 2019; Grunert et al., 2019). Data were imported into R using phyloseq, and taxon abundances were rescaled by calculating the taxon proportions and multiplying them by the minimum sample size (*n* = 2,210) present in the data set (Hernandez-Sanabria et al., 2020). Taxon data were used, and if any OTU was not classified up to a family level, the consensus sequence was blasted using the NCBI database, and taxonomic classification was obtained. Species richness, diversity (inverse Simpson index), and evenness (Pielou’s index) of horticultural growing medium were calculated within each sample to elucidate the effect of the plant presence, sterilization, fertilizer type, location (root vs. bulk), and time (Grunert et al., 2019). Beta-diversity estimates based on Chao and Bray–Curtis indices were used to examine community dissimilarity and to determine the impact of experimental factors on the microbial community structure. PCoA was employed to visualize the differences among samples, using the vegan package in R. Stratified PERMANOVA with 999 permutations was conducted to indicate the significance of each covariate (time or fertilizer) on the microbial community (Hernandez-Sanabria et al., 2020).

### Evaluating Relationships Between Microbial Community Characteristics and Physicochemical Characteristics

Beta diversity was used to examine if the different fertilizers have an impact on the microbial community structure. Principal coordinate analysis (PCoA) was employed to visualize the differences among samples (Supplementary Figure 1). Bipartite networks highlighted functional associations among bacterial genera and physicochemical characteristics of the rhizosphere of the growing medium. Bipartite networks were inferred using a similarity matrix obtained from a Regularized Canonical Correlation Analysis (rCCA), using the package mixOmics in R (Lê Cao et al., 2011). In this analysis, the values in the similarity matrix were computed as the correlation between the relative abundances of bacterial genera and physicochemical characteristics of the growing medium and plant outcomes. Then, these values are projected onto the space spanned by the first components retained in the analysis. Four relevant components were obtained setting a threshold to *r* = 0.85. Relevance networks are a robust approach to highlight functional relationships, as a result of their ability to simultaneously represent positive and negative correlations, which are missed by methods using Euclidian distances or mutual information. Another advantage of the rCCA is its ability to represent correlations across disparate biological measures, such as the bacterial relative abundances and physicochemical characteristics of the growing medium. Genera in the plot were close to correlated variables in the treatment where they were more abundant (Hernandez-Sanabria et al., 2020). Differences in the relative abundance of OTUs representing the genus *Rhizobium* sp. were tested using DESeq2 (v1.18.1) with default settings while controlling false discovery rate (FDR) using the Benjamini–Hochberg correction for an adjusted *P* < 0.01. This analysis supports our hypothesis that there are functional relationships among members of the bacterial community, preserved across fertilizers and others that are observed only on a particular fertilizer.

### Analyses of Variance

Measurements of shoot biomass, pH, nutrient content in plant and soil, and root morphological traits were analyzed with two-way analysis of variance (ANOVA). Tukey’s HSD *post hoc* test after ANOVAs at a significance level of *p* < 0.05 was used to see which level of a factor differs from one another. Data were calculated as arithmetic means ± standard error of the mean of the indicated replicates.

## Results

### Shoot Biomass Remained Consistent Across Fertilizer Treatments

The measures in lupine dry weight at time point 1 (14 DAS) showed no differences among any of the N treatments, with an average of 0.14 g for struvite and organic fertilizer treated plants, and 0.13 g for control (no N application) plants. Only in the second harvest, i.e., at time point 2 (27 DAS), plants treated with organic fertilizer had a 50% higher dry weight (average 0.66 g) than struvite or no N (average 0.42 g) (Table 3); however, no significant differences (*P* < 0.05) could be determined. This indicates that N source applied did not have a significant impact on plant growth at the experimental conditions applied in our study.

**TABLE 3:** Influence of fertilizer type: no fertilizer, organic fertilizer, and struvite on plant performance of lupine in the organic growing medium in function of time.

Variable	Tpt	Fertilizer		
		NoF	ORG	STR
Leaf area (cm^2^)	0	NA	NA	NA
	1	23.9*c*2.3	25.7*c*2.1	22.4*c*2.1
	2	52.9*bc*3.7	86.9*b*2.7	65.6*b*9.03
Fresh weight (g)	0	NA	NA	NA
	1	1.11*b*0.1	1.25*b*0.1	1.24*b*0.1
	2	2.58*b*0.1	4.5*b*0.1	3.36*b*0.5
Dry weight (g)	0	NA	NA	NA
	1	0.13*b*0.02	0.145*b*0.03	0.143*b*0.01
	2	0.408*b*0.02	0.667*b*0.02	0.423*b*0.07

**Values are mean (n = 5) ± SD. Tpt 0 = sowing day (0 DAS), tpt1 = time point 1 (harvest 1, 14 DAS) and tpt 2 = time point 2 (harvest 2, 27 DAS). NA, not available. Different letters indicate significant differences. GB without fertilizer = NoF; GB with organic fertilizer = ORG; GB with struvite = STR.**

### pH Changes and Nitrogen Turnover in the Growing Medium Fluctuated Depending on the Source of N Applied

Total N added at the beginning of the experiment was the same with both fertilizers (100 mg N L^–1^); however, the nitrogen form was different, i.e., ammonium for the struvite and organic N for the organic fertilizer. Ammonium and nitrate concentrations (mg L^–1^) in the growing medium of lupine were analyzed on the sowing day (time point 0). As expected, the concentration of ammonium measured on the growing medium was higher after the addition of struvite than after the addition of organic fertilizer (*P* < 0.001, Table 4), although this did not necessarily mean that this ammonium was directly available. The concentration when no fertilizer was applied was the same as the initially measured in the growing medium analyses (1.7 mg NH_4_^+^ L^–1^, Table 4).

**TABLE 4:** Influence of fertilizer type (no fertilizer—NoF, organic fertilizer—ORG, and struvite—STR) on the nutrient dynamics and pH in non-sterile organic growing medium with plants (lupine) and without plants (no plant, as control) in function of time.

Time point	Species	Fertilizer	NH_4_-N (mg L^–1^)	NO_3_ -N (mg L^–1^)	P0_4_-P (mg L^–1^)	pH
Starting exp.	No plant yet	NoF	1.71.4	0.01.03	18.74.9	5.50.03
		ORG	18.41.4	0.01.03	27.34.9	5.70.03
		STR	42.11.4	0.01.03	203.14.9	5.60.03
First harvest	No plant	NoF	3.61.4	0.01.03	12.94.9	6.20.03
		ORG	20.11.4	2.51.03	21.64.9	6.20.03
		STR	49.41.4	0.01.03	150.14.9	6.10.03
	Lupine	NoF	5.81.4	0.01.03	12.44.9	6.70.03
		ORG	22.81.4	0.01.03	25.34.9	6.30.03
		STR	70.11.4	0.01.03	224.74.9	6.10.03
Second harvest	No plant	NoF	0.41.4	0.01.05	11.55.0	6.00.03
		ORG	16.31.4	13.81.05	19.75.0	5.60.03
		STR	54.71.4	13.01.05	206.15.0	5.60.03
	Lupine	NoF	1.61.4	0.01.05	29.55.0	6.10.03
		ORG	3.01.4	36.41.05	23.35.0	5.30.03
		STR	50.71.4	26.91.05	331.05.0	5.40.03

**Values are mean (n = 5) ± SEM (P < 0.05). The organic growing medium (GB) without fertilizer = NoF; GB with organic fertilizer = ORG; GB with struvite = STR.**

The ammonium concentration (measured via water extraction) in the growing medium with lupine but without N fertilizer accounted for 5.8 mg L^–1^ at harvest 1 (14 DAS), indicating a small increase of 4 mg L^–1^. This increase was only up to 2 mg L^–1^ in the growing medium unfertilized and without plants. At this time point, the concentration of ammonium with organic fertilizer was 22 mg L^–1^ and 70 mg L^–1^ with struvite (Table 4). No nitrate was measured in the growing medium with plants at this point; however, the growing medium without plants treated with the organic fertilizer showed an increase of 2 mg nitrate L^–1^ growing medium. The ammonium concentration in the growing medium with lupine was reduced around 20 mg L^–1^ from harvest 1 to harvest 2 (27 DAS) in both N treatments. The nitritation and subsequent nitratation of ammonium to nitrate were higher in the organic fertilizer, with a nitrate concentration of 36 mg L^–1^, compared to the 27 mg L^–1^ measured in the struvite treatments. These results indicate that lupine plants are capable of rapidly releasing ammonium from the struvite and successfully performing nitrification, but more effective nitrification of ammonium to nitrate from organic fertilizer.

Plant (*P* < 0.005, 2-way ANOVA/Tukey HSD) and time (*P* < 0.001, 2-way ANOVA/Tukey HSD), but not fertilizer, had a significant effect on the pH of the growing medium. The overall pH (H_2_O) was 5.6 ± 0.03 at the start, increased to 6.2 ± 0.03 at time point 1 and decreased again to 5.7 ± 0.03 at the second time point. Organic fertilizer and struvite resulted in similar pH changes in the growing medium (Table 4). Under no fertilizer, the increase of the pH was the highest (up to 6.7). The initial increase of pH was also observed, to a lesser extent, with no plants, indicating that plants had a more significant effect than the fertilizer applied on the pH of the growing medium (*P* < 0.005).

### Plant Nutrient Content Is Independent of the Nitrogen Source and Directly Associated With Accelerated Plant Growth

Shoot nutrient concentrations of lupine were compared between struvite and organic fertilizer applied as N sources. Struvite led to a higher but not statistically significant concentration of N (6 vs. 5.3% organic in the first harvest and 4.4 vs. 3.9% organic in the second harvest, Table 5). The only treatment that showed concentrations within the N deficiency levels, i.e., less than 3% N as indicated by Marschner for legume representative species (Marschner, 2011), was the no fertilizer treatment at time point 2. Phosphorus shoot concentration with struvite was significantly higher than with organic fertilizer at both time points (0.79% struvite and 0.62% organic fertilizer at second harvest; however no significant differences were observed in the total P uptake (*P* < 0.05, Table 5). There were no significant differences in the shoot concentrations of K and Mg between treatments, but the K uptake in the organic fertilizer treated plants was higher than with the struvite.

**TABLE 5:** Nutrient uptake and N recovery in the plant.

	Harvest	Total N uptake (mg)	N shoot conc. (% DW)	% plant N recovery*	Total P uptake (mg)	Total K uptake (mg)	Total Mg uptake (mg)
NoF	1	7.00.8	5.10.6	na	0.50.1	4.11.04	0.70.1
ORG	1	7.81.7	5.40.3	0.20.3	0.90.2	4.30.9	0.70.2
STR	1	8.60.8	6.00.3	0.30.2	1.10.1	3.60.2	0.90.08
NoF	2	8.60.9	2.10.2	na	1.20.1	12.90.5	2.70.2
ORG	2	25.40.9	4.20.1	3.40.2	3.80.1	19.40.7	4.00.2
STR	2	21.53.3	4.50.1	2.60.7	3.80.7	13.51.8	3.50.5

**The organic growing medium (GB) without fertilizer = NoF; GB with organic fertilizer = ORG; GB with struvite = STR. DW = dry weight. Values are mean (n = 5) ± standard deviation.**

N recovery indicates the % of N taken up by the plant and was calculated as indicated in F1 (taking into account the N uptake under unfertilized conditions due to N fixation). N recovery from struvite and organic fertilizer in the first harvest was very low (0.16 and 0.32%, respectively). This value increased in the last harvest to a maximum of 3.3% from the organic and 2.5% from the struvite. Still, this small N recovery made a significant difference in the N shoot concentration compared to the unfertilized plants (around 4% for the fertilized plants and 2% for the non-fertilized plants).

It was observed that d^15^N vs. air (‰) was the smallest in the no fertilized plants (0.05‰) in comparison with the fertilized plants (3‰), indicating that no fertilized plants increase the N fixation in comparison to the fertilized plants (Table 6).

**TABLE 6:** d^15^N analyses in lupine shoot at final harvest.

	Harvest	N content (%)	d^15^N vs. air (‰)	Max. diff. between N
NoF	2	2.24 ± 0.1	0.05 ± 0.2	0.01 ± 0.01
ORG	2	4.19 ± 0.09	3.16 ± 0.2	0.03 ± 0.01
STR	2	4.53 ± 0.1	3.00 ± 0.1	0.02 ± 0.01

**The organic growing medium without fertilizer = NoF; GB with organic fertilizer = ORG; GB with struvite = STR. Values are mean (n = 3) ± standard deviation.**

### Nitrogen Release From Organic Fertilizer Was Moved to Another System; However, Most of the N Released From Struvite Remained Immobilized in the Growing Medium

To analyze the N balance, the % of mineral N (N_min_) that remained in the growing medium and the % of N measured in the plant related to the N applied were calculated (Table 7). In the struvite treatment, most of the N applied (up to 78%) remained in the growing medium at the end of the experiment, while a small percentage was taken up by the plant (2.5%) and around 17% was not measured. With the organic fertilizer treatment, only around 40% of the N applied remained in the soil at the final harvest in the form of N_min_. A small percentage was recovered by the plant (3.3%), but more than 55% of the applied N was not measured. These results might indicate that the N from the organic fertilizer was still in the form of organic N or was used by another system, such as the native microbial community, or it was lost to the atmosphere hindering its quantification in the soil. Besides the differences in the N turnover, there were no significant differences in the plant growth between fertilizers.

**TABLE 7:** Analyses of nitrogen balance.

	Harvest	% N measured in the soil vs. N applied	% N measured in plant+soil vs. N applied
NoF	1	Na	na
ORG	1	22.785.1	24.344.8
STR	1	70.1013.3	71.8213.3
NoF	2	Na	na
ORG	2	39.406.4	44.496.5
STR	2	78.768.5	83.068.2

% N measured in the soil vs. N applied stand for the amount of N applied that is measured at each harvest in the soil (total. ammonium and nitrate) in relation to the N applied. Total % measured indicates the % of N applied that is measured combining the total N in the soil and the plant tissue for each replicate. Each number is the mean value of n = 5 ± standard deviation. The organic growing medium without fertilizer = NoF; GB with organic fertilizer = ORG; GB with struvite = STR. na: not available, as no N was applied. N recovery = (N plant fert − N plant unfert) * 100/N applied.

### Comparable Root Growth Between Struvite and Organic Fertilizer Might Be Related to Similar N Decomposition in the Growing Medium

Plants were grown in growing medium-filled rhizotrons, allowing for simultaneous quantitative measurements of root architecture parameters and shoot biomass in 2D, over time.

**FIGURE 1:**
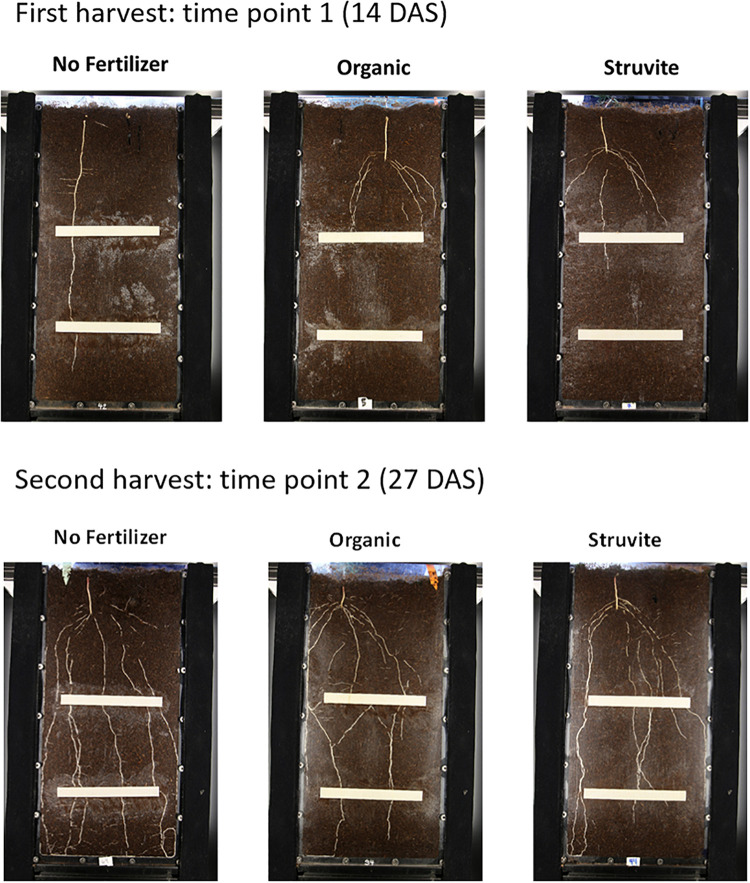
Rhizotron of 60 cm × 30 cm × 2 cm filled with organic substrate and planted with lupine. Visible roots are observed at harvest 1 (14DAS) and harvest 2 (27 DAS) for the three treatments (no fertilizer, organic fertilizer, and struvite).

**FIGURE 2:**
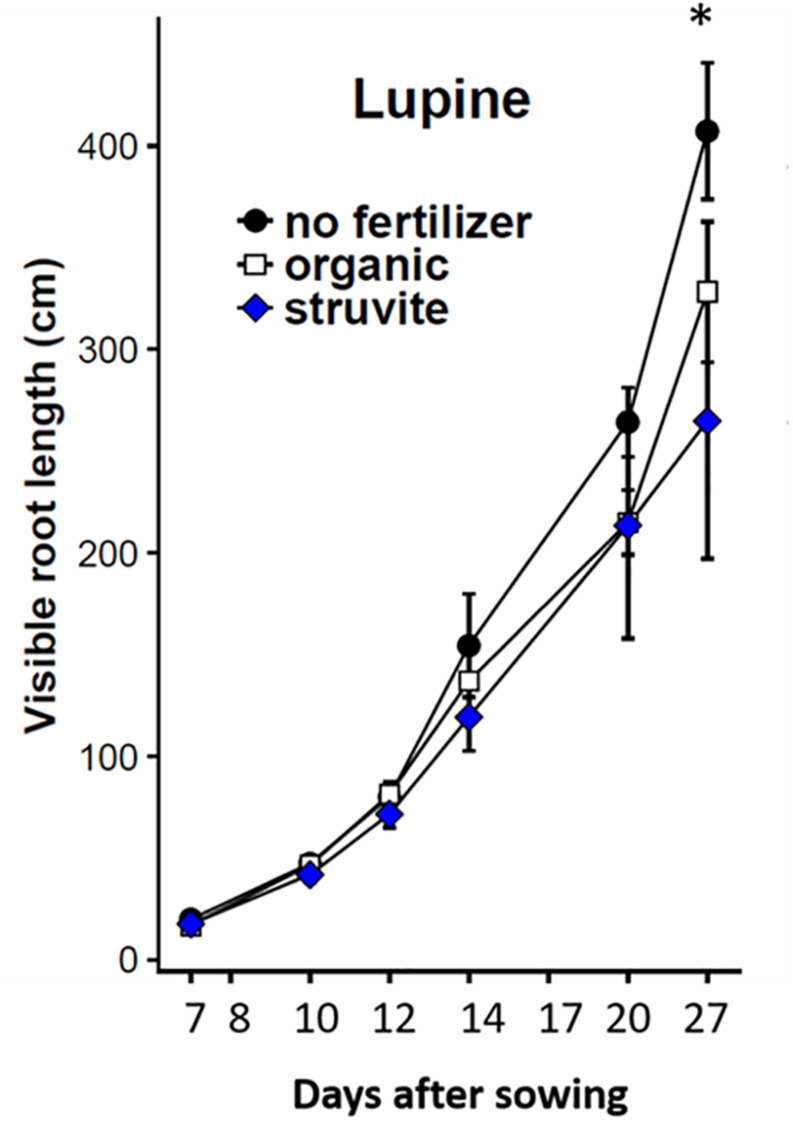
Total root length (cm) of lupine growing in rhizotrons filled with the organic substrate as affected by fertilizer applied (no fertilizer, organic or struvite). Non-invasive measurements were done at different time points indicated in the *X*-axis as days after transplanting. Points are averages of *n* = 5 ± standard error of the mean. * Indicates significant differences between no fertilizer and organic/struvite fertilization (*p* < 0.05, one-way ANOVA).

The percentage of visible roots (∼20–30% visually estimated in this experiment) decreases with an increasing average root diameter of the plant species studied and depends, to some extent, on environmental conditions (Nagel et al., 2012). We recorded changes in root architecture at different stages of root development of lupine plants (Figure 1). Higher root length was observed when no fertilizer was applied (Figure 2). A similar distribution of primary-thicker roots and secondary-thinner roots between organic and struvite fertilizers was observed (Supplementary Table 1). There were no significant differences in root growth between plants at time point 1 (*P* < 0.05); however, fertilized plants had a reduced length of the taproot compared with the unfertilized plants, where the taproot grew faster reaching the bottom of the rhizotron earlier. Fertilized plants increased the number of thinner lateral roots during the first measurement days (measurement day 1–3) in comparison to the no-fertilized plants (Supplementary Table 1). At time point 2 (measurement day 6), no fertilized plants had a higher total root length compared to the fertilized ones.

### Interplay Between the Microbial Community and Physicochemical Characteristics Indicated Dynamic Adaptations to N Sources

Community metrics in the rhizosheath at the first harvest were significantly different as a result of the fertilizer treatment. Microbial richness, evenness, and overall alpha diversity were higher when no fertilizer was supplied. However, these metrics were not significantly different at the second harvest. This may indicate that time plays an essential role in the bacterial colonization of the rhizosheath of lupine plants (*P* < 0.05, Supplementary Figure 2). Community metrics of the rhizosphere were not significantly different as a result of the fertilizer used, plant presence, and growing medium pretreatment, i.e., sterile vs. non-sterile. Microbial community evenness tended to increase over time in the rhizosphere, confirming the impact of time on the bacterial community in the growing medium harboring lupine plants. On the contrary, evenness in the rhizosheath remained constant over time. Communities with high evenness show higher functional stability even under non-stressed conditions. Thus, the increase in evenness may indicate that communities become more stable at the second harvest. The lower variability among samples of growing medium supplied with struvite suggests that the communities exposed to this fertilizer are always stable. Permutational multivariate analysis of variance (PERMANOVA) confirmed that time point and fertilizer equally and significantly contributed to the differences in the mean relative abundances of bacterial genera (*P* < 0.001) among rhizosheath communities (Supplementary Figure 1A). Time and soil pretreatment significantly impacted the mean bacterial relative abundances of the communities in the rhizosphere (Supplementary Figure 1B). Bacterial communities were dissimilar at the start of the experiment, in comparison with those at the end of the second harvest, where communities exposed to organic fertilizer were significantly different.

**FIGURE 3:**
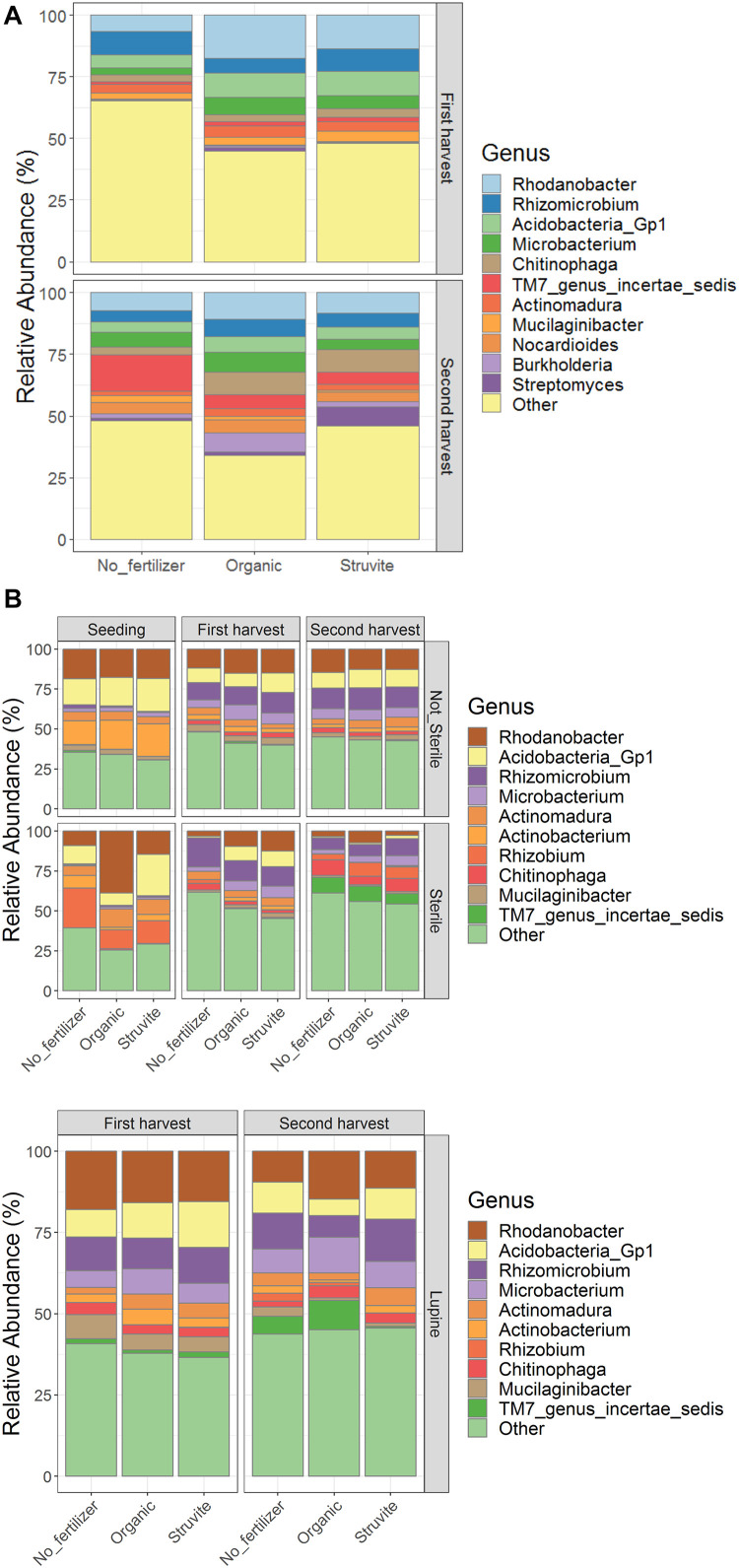
The relative abundance of bacterial genera shifted over time with fertilizer application in the rhizosheath **(A)** while pretreatment of growing medium impacted communities in the rhizosphere (**B**, upper panel). Rhizosphere of growing medium hosting lupine plants (**B**, lower panel) showed similar community composition.

We identified spp. belonging to *Rhodanobacter*, *Rhizomicrobium*, *Acidobacteria*, *Microbacterium*, *Chitinophaga*, *Actinomadura*, *Mucilaginibacter*, *Nocardioides*, *Burkholderia*, *Streptomyces*, and *TM7 genus incertae sedis* as the bacterial genera with the highest relative abundance in the rhizosheath (Figure 3A). The relative abundance of the N-cycle *Rhizomicrobium* decreased over time in the presterilized growing medium regardless of fertilizer applied, while *Actinomadura* increased in the first harvest in the growing medium without fertilizers (Figure 3B, upper and lower panels). *Burkholderia* and *Nocardioides* replaced *Actinobacterium* and *Rhizobium* in the rhizosheath (Figure 3A). Oligotrophic microorganisms such as *Acidobacteria* decreased their relative abundance over time. These results suggest that the rhizosheath-associated bacterial community evolved into copiotrophic populations as a result of the increased N available in the growing medium.

**FIGURE 4:**
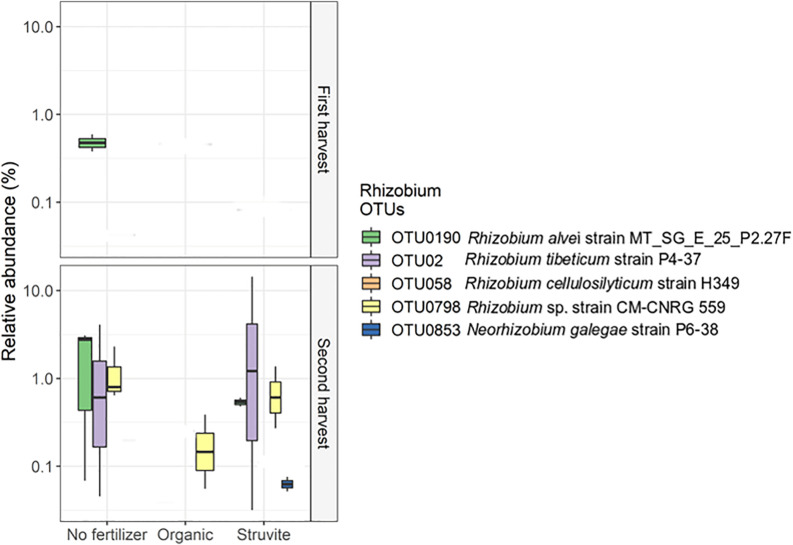
High phenotypic diversity between representatives of the genus *Rhizobium* inhabiting the rhizosheath of lupine plants supplemented with different fertilizers. The copiotrophic *Rhizobium* showed a decreased relative abundance in the rhizosheath. Five different OTUs represented this genus, and OTU02, OTU0190, and OTU0798 displayed significant variation on their relative abundances. The sequences of these OTUs were blasted on the NCIBI nucleotide search engine and yielded the following: OTU02 was 100% similar to the endophytic *Rhizobium tibeticum* strain P4–37, OTU0190 was 99.5% similar to *Rhizobium alvei* strain MT_SG_E_25_P2.27F, and OTU0798 was 99.5% similar to *Rhizobium* sp. strain CM-CNRG 559 (found in chickpeas). Please note that the orange OTU is too low in abundance to show on the graph.

*Rhizobium* showed low abundance in the rhizosheath. Five different OTUs represented this genus, and OTU02, OTU0190, and OTU0798 displayed significant variation on their relative abundances (Figure 4). The sequences of these OTUs were blasted on the NCBI nucleotide search engine and yielded the following: OTU02 was 100% similar to the endophytic *Rhizobium tibeticum* strain P4-37, OTU0190 was 99.5% similar to *Rhizobium alvei* strain MT_SG_E_25_P2.27F, and OTU0798 was 99.5% similar to *Rhizobium sp.* strain CM-CNRG 559 (found in chickpeas). These results indicate high phenotypic diversity between representatives of the genus *Rhizobium* inhabiting the rhizosheath of lupine plants. Moreover, analysis of the significant differences in taxa abundance uncovered a significant log2 fold difference in *Phenylobacterium* in growing medium supplemented with an organic fertilizer when compared with medium without fertilizer.

**FIGURE 5:**
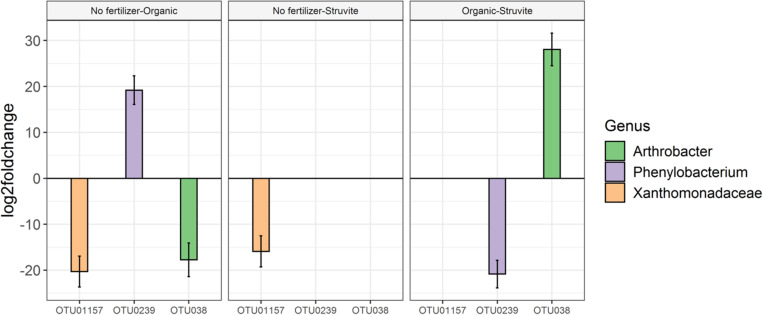
Analysis of the significant differences in taxa abundance uncovered a log2 fold increase in *Phenylobacterium* in growing media supplemented with an organic fertilizer when compared with medium without fertilizer. No difference was found between the no fertilizer treatment and struvite; consequently, this genus was significantly less abundant when struvite was applied and compared with the organic fertilizer (*P* < 0.05, Figure 5). *Arthrobacter* was significantly more abundant in the growing medium supplied with organic fertilizer, in comparison with struvite.

**FIGURE 6:**
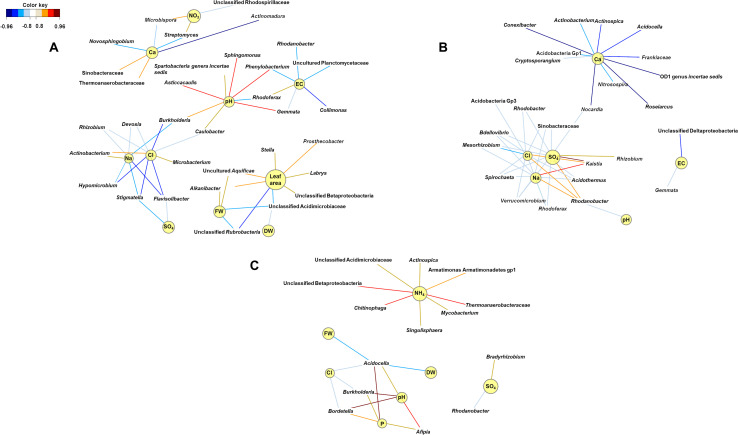
Community metabolic activity indicates that networks of bacterial interactions were more numerous when struvite was supplied **(A)** and decreased with organic fertilizer **(B)** and when fertilizer was not provided **(C)**. Struvite seemed to promote associations between potentially beneficial endophytes and plant characteristics. These bipartite networks are based on the regularized canonical correlations between relative bacterial abundances and physicochemical characteristics of the growing medium and plant outcomes. Interactions have been filtered for an absolute correlation above 0.85 and are colored following the key shown. Significant interactions are indicated by shorter lines, and genera with similar abundances within treatment tended to cluster closely.

Relevance network analyses performed at the second harvest indicated that genera interacting with the physicochemical characteristics of the growing medium shifted with fertilizer. *Phenylobacterium* was significantly abundant when struvite was provided (*P* < 0.05, Figure 5), and it was positively associated with pH (*P* < 0.05, Figure 6A). Similarly, *Sphingomonas*, *Asticcacaulis*, and *Gemmata* were associated with pH. The genus *Streptomyces* was positively associated with ammonium, while the copiotrophic *Actinobacterium* was associated with chlorides (*P* < 0.05, Figure 6A). Electrical conductivity, pH, Na, and Cl were variables interrelated in the struvite network, suggesting that the bacterial community increased their metabolic activity when struvite was applied. *Rhizobium* tended to be associated with sulfates when the growing medium was supplied with organic fertilizer (*P* < 0.05, Figure 6B), while *Mesorhizobium* was negatively associated with chlorides. *Rhodanobacter* was positively associated with sulfates, Na, and Cl but negatively associated with pH (*P* < 0.05). These results are confirming its reported presence in low pH soils (Kostka et al., 2012). *Burkholderia*, *Bordetella*, and *Afipia* were positively associated with pH in growing medium without fertilizer (Figure 6C). *Chitinophaga*, Thermoanaerobacteriaceae, and unclassified Betaproteobacteria were significantly associated with ammonia (*P* < 0.05), while *Bradyrhizobium* tended to be associated with higher sulfates. The smaller number of networks observed when fertilizer was not supplied may indicate low activity of the bacterial community. Our network analysis revealed that the treatment with struvite yielded the highest number of associations with all plant variables (leaf area, fresh, and dry weight). This may imply enhanced operational efficacy of the bacterial populations on the growing medium when struvite is provided, ultimately impacting plant outcome.

## Discussion

Plant N recovery (% of the applied N that is taken up) was higher from the organic fertilizer than from struvite, indicating that N from the organic fertilizer was more easily available to plants, contrary to what we hypothesized. The nutrient content of a plant not only varies among its various tissue parts but also changes with age and stage of development. Therefore, analyzing nutrient concentration rather than content might allow for a more precise diagnosis of plant nutritional state. Nutrient deficiency or toxic values are typically described in % of dry weight ranges (Marschner, 2011). For leguminous plants, such as lupine, the critical N concentration is less than 3% (Marschner, 2011). The N concentration in lupine was at the expected level (from 3.9 to 6%), with no significant differences between fertilizers. This indicates that lupine plants fulfilled their N needs under both fertilizer treatments.

N recovery from struvite and organic fertilizer was very low, but still, this small N recovery made a significant difference in the N shoot concentration and plant biomass compared to the unfertilized plants. The small recovery might be explained by the high fertilization rate and the short period of the experiment (27 DAS). Furthermore, nitrogen concentration in shoots decreased during growth, as indicated by the lower N concentration in lupine plants in the second harvest. This can be explained as the N uptake is assumed to be lower than the crop growth rate. Also, N concentration decreases with plant growth as there is a higher amount of metabolic tissues with higher nitrogen concentration in the juvenile plant stage (Marschner, 2011). Our results indicate that physiological changes may play a secondary role in the reduction of the concentrations of nitrogen in plant tissue at the second harvest. The efficiency of nitrogen use by the lupine is probably a size-dependent phenomenon resulting from the accelerated plant growth.

Struvite was compared with an organic fertilizer made of organic matter that first required breakdown into amino acids, whose application as a fertilizer has been demonstrated to have a beneficial effect on leaf mineral status (Garcia et al., 2011). It was hypothesized that struvite would have a beneficial effect as N source, as it delivers the ammonium directly in the rooting medium. On the other hand, the organic fertilizers need a supplementary conversion first from the organic N to ammonium to be plant available.

Trying to demonstrate this, the calculation of N balance in the plant-growing medium was performed. The ammonium concentration in the growing medium with lupine but without fertilizer had an increase of 4 mg L^–1^ in the first harvest, 2 mg L^–1^ more than what was measured in the growing medium unfertilized and without plants. Next to other factors, watering of a growing medium stimulates the release of nutrients through decomposition and mineralization, explaining the increased ammonium concentrations in the non-fertilized setup without plants. The higher ammonium concentration in the medium with plants but without fertilizer might be explained by increased microbial activity due to root C inputs (Clarholm, 1985) or decreased microbial immobilization resulting from more effective competition for N by plants (Griffiths and Robinson, 1992). At this time point, the concentration of ammonium with organic fertilizer was 22 and 70 mg L^–1^ with struvite (Table 4). Even though the ammonium from the struvite in the growing medium was indeed higher compared to the organic fertilizer, this was not reflected in a higher N uptake or higher biomass. The oxidation of ammonium to nitrate in the second harvest was higher in the organic fertilizer treatment, indicating that lupine plants associated bacteria are more effective mineralizing ammonium from organic fertilizer but also still can successfully perform nitrification from struvite, contrary to what was observed in previous experiments with other species such as tomato (Grunert et al., 2019).

PH changes might be related to the initial ammonium dissolved from the struvite and organic fertilizer that is still present in the growing medium and not taken up by the plant. Under no fertilizer conditions, the increase of the pH was the highest (5.5–6.7). This might be explained by the liming effect of [(Ca, Mg)CO_3_]_2_, which was added to the growing medium to adjust the pH to the desired level.

The use of rhizotrons allowed us to analyze root growth over time and to observe changes in root architecture at different stages of root development. The non-fertilized plants had higher total root length than the fertilized plants, independently of the N source. This might be explained by the nutrient content in the seed and the successful nodulation observed in the no N treatment that provided the plant with the extra N needed to establish the seedlings and initial plant growth. Moreover, it was observed that these non-fertilized lupines increased primary root growth and decreased the lateral root density, contrary to what was observed in the fertilized plants. These variations in the root morphology might affect the percentage of visible roots in the rhizotrons. Usually, this value is ∼20%, but it decreased with an increasing average root diameter of the plant species (Nagel et al., 2012). That might explain the final higher root length measured in lupine plants under no fertilizer (with higher root diameter, i.e., higher % of visible roots) compared with the secondary-thinner roots in organic fertilizer and struvite treatments.

In a previous study with tomato (Grunert et al., 2019), organic fertilizer led to a higher root length compared to struvite. In that case, the rhizosphere microbial community of tomato plant was not able to mineralize the ammonium from the struvite, which entailed significant differences in the ammonium concentration between both fertilizers in the growing medium that probably affected root architecture (Liu and von Wirén, 2017). In our study, lupine plants were able to mineralize ammonium from struvite in a similar way than from the organic fertilizer. It is known that the root morphology of lupine is modulated by the N source (ammonium or nitrate) present in the soil (Robles-Aguilar et al., 2019b). Therefore, similar concentrations of ammonium and nitrate, in this case, could be related to the similar root morphology of lupine under both N fertilization regimes. However, this explains only a part of the difference between lupine and tomato, as it seems that plant species also have a big impact on the root/shoot ratio.

Struvite treated growing medium showed in general 10–20 times higher P concentrations compared to the organic fertilizer and the no fertilizer treatment. According to preceding research, under low plant-available phosphorous concentrations, nodules would mainly decrease the utilization of atmospheric nitrogen as primary nitrogen source and utilize more readily plant-available nitrogen sources such as nitrate and ammonium (Valentine et al., 2017). Furthermore, root nodulation is accelerated by low concentrations of nitrogen and significantly suppressed by high concentrations of nitrogen. Minchin and Witty (2005) showed that nitrate itself is a strong inhibitor of nodulation and hence nitrogen fixation but also that the utilization of carbon as an energy source for nitrogen uptake from the soil is less than that for nitrogen fixation. That might explain why N fixation was not the preferred path to take up nitrogen in the fertilized plants.

Ammonium availability from a fertilizer might be related not only to specific plant nutrient turnover strategies or to the form that the nitrogen is delivered in (organic or inorganic) but also to other factors like the microbial activity. The activity is associated with each species that might prefer one source of N over another, affecting the nitrification of ammonium differently regarding the N source (Grunert et al., 2016b, 2019). The active, competitive and resilient native community has been observed in the type of organic growing medium used in this experiment. Previous work from our team reported that tomato plants modify the structure and function of the bacterial community rather than the applied fertilizer (Grunert et al., 2019). We hypothesized that this would also be the case with lupine plants. However, fertilizer had a significant impact on the associations and functionality of the bacteria inhabiting the growing medium used, suggesting that fertilizer influenced the interindividual dissimilarities in the most abundant genera between treatments. As symbiotic bacteria support legumes in meeting their nutrient demands (Velázquez et al., 2017), less competition for nutrients between lupine and its bacterial community potentially occurred in our study.

Microbial richness, evenness, and diversity were significantly higher in the rhizosheath at the first harvest when no fertilizer was supplied; however, these metrics were not significantly different at the second harvest and in the rhizosphere. No differences were found between the organic N and the inorganic N source. Time and soil pretreatment significantly impacted the mean bacterial relative abundances of the communities in the rhizosphere (Supplementary Figure 1B). Bacterial communities were dissimilar at the start of the experiment, in comparison with those at the end of the second harvest. This may indicate that time plays an essential role in the bacterial colonization of the rhizosheath of lupine plants. Indeed, the structure and function of the microbial communities in the rhizosphere are cooperatively orchestrated by plant and growing medium (Berg and Smalla, 2009). These results are in agreement with earlier research showing reduced biodiversity upon N supplementation (He et al., 2007; Geisseler and Scow, 2014; Ling et al., 2017; Zhou et al., 2017); however, earlier-mentioned research studied the long-term effects of inorganic N sources, while in our study short-term and inorganic and organic N supplementations were examined. The structure of the rhizosphere associated microbial community is gardened by a complex trade of compounds between the microorganisms and the plant (Werner et al., 2014), which can have plant growth-promoting effects for the plants (Pii et al., 2015). Indeed, microorganisms can alter nutrient availability in the rhizosphere (Pii et al., 2015). Differences in the microbial community structure of the rhizosphere were mainly a result of time, which has been described for maize (Baudoin et al., 2002), tomato (Grunert et al., 2019) and now also for a legume. Therefore, we concluded that the change of microbial community structure in the rhizosheath of lupine could be attributed to the direct influence of plant rather than the fertilizer.

We identified *Rhodanobacter*, *Rhizomicrobium*, *Acidobacteria*, *Microbacterium*, *Chitinophaga*, *Actinomadura*, *Mucilaginibacter*, *Nocardioides*, *Burkholderia*, *Streptomyces*, and *TM7 genus incertae sedis* as the bacterial genera with the highest relative abundance in the rhizosheath (Figure 3A). The relative abundance of the N-cycle *Rhizomicrobium* increased over time, regardless of fertilizer applied, while *Actinomadura* increased in the growing medium supplemented with fertilizers. *Actinobacterium* and *Rhizobium* replaced *Burkholderia* and *Nocardioides* in the rhizosphere (Figure 3B), regardless of plant presence. *Acidobacteria* are one of the most general and abundant phyla on earth (Kielak et al., 2016). Cultured *Acidobacteria* are heterotrophic, they can use multiple carbon sources, and they are able to use nitrite as a nitrogen source. However, there is no clear proof for the role of *Acidobacteria* in N-cycle processes (Kielak et al., 2016). We reported positive associations between ammonium and *Streptomyces* in the rhizosphere when struvite was supplied. Plant growth-promoting rhizobacteria (PGPR), such as *Burkholderia*, can enhance nutrient acquisition through nitrogen fixation, phosphate solubilization, sulfur oxidation, and iron acquisition. Different *Streptomyces* strains, such as *Streptomyces thermoautotrophicus* (Ribbe et al., 1997), showed plant growth-promoting effects in *Arabidopsis* (Cordovez et al., 2015), rice, wheat, sorghum, and tomato (Gopalakrishnan et al., 2013, 2014). *Rhodanobacter* is a genus known to reduce nitrate, playing a key role in the nitrogen cycle (Kostka et al., 2012). We observed that ammonium nitrogen concentration was significantly high both in the rhizosphere and in the rhizosheath. This variable may have been correlated with the relative abundance of *Rhodanobacter*. Indeed, *Rhodanobacter* was positively associated with sulfates, Na, and Cl when organic fertilizer as applied and with low EC when struvite was applied. For this reason, *Rhodanobacter* may be one of the main genera impacting pH in the growing medium, regardless of fertilizer supplementation. For this reason, *Rhodanobacter* may be one of the main genera impacting pH in the growing medium, regardless of fertilizer supplementation. These results suggest that the rhizosheath-associated bacterial community evolved into copiotrophic populations (Ho et al., 2017) as a result of the increased N and carbon sources available in the growing medium (Fierer et al., 2012; Ramirez et al., 2012; Männistö et al., 2016).

*Rhizobium* showed a decreased relative abundance in the rhizosheath. Five different OTUs represented this genus, and OTU02, OTU0190, and OTU0798 displayed significant variation on their relative abundances (Figure 4). These results confirm the high phenotypic diversity between representatives of the genus *Rhizobium*, known for its nitrogen-fixing capabilities (Beijerinck, 1901), inhabiting the rhizosheath of lupine plants supplemented with different fertilizers. Additionally, *Arthrobacter*, a heterotrophic nitrifier (Verstraete and Alexander, 1973), was significantly abundant when organic fertilizer was supplied. This genus can inhibit the growth of phytopathogenic fungi and enhance salt tolerance in plants (Velázquez-Becerra et al., 2013). We confirmed that *Xanthomonadaceae* OTUs were significantly more abundant in rhizosheath supplied with struvite and organic fertilizer. Endophytic *Xanthomonas* ribotypes have been recorded in *L. angustifolius* nodules (Ferchichi et al., 2019). PGPRs such as *Sphingomonas* and *Burkholderia* (Compant et al., 2005; Khan et al., 2014, 2017) and *Phenylobacterium* (Lingens et al., 1985), a Gram-negative bacterium that degrades xenobiotic compounds, were positively associated with pH when struvite was supplied. Strains of rhizobia can gain access through cracks from new roots and not only colonize the roots but also migrate upward into the stem base, leaf base, leaf sheaths, and some leaves of the plant (Chi et al., 2005). We detected that *Prosthecobacter*, which use ammonium as the preferential nitrogen source (Takeda et al., 2008), *Labrys* (Inui et al., 2012), and uncultured *Aquificae* were positively associated with leaf area when struvite was supplied. *Alkanibacter* was also associated with this plant variable. Our results suggest that PGPR and other bacteria present in the rhizosheath may migrate to the root nodules of lupine later in life.

Our study addresses some limitations of previous studies and extended our knowledge about the effect of applying recovered nutrients (such as organic fertilizer and struvite) in growing media on belowground microbiology. To do that, (1) rhizosphere and rhizosheath samples were studied in addition to bulk growing media samples; (2) the bacterial community was assessed using Illumina sequencing; and (3) rhizotrons were used to test their effects on root length and morphology, plant performance, and nutrient recovery in lupine plants.

Compared with aboveground plant parts, roots are not easily accessible by non-invasive analyses and research is still based mainly on destructive methods at harvest. Plants were grown in growing medium-filled rhizotrons, allowing for simultaneous quantitative measurements of root architecture parameter and shoot biomass in 2D over time, and rhizotrons are helpful instruments for guided accurate sampling. Still, a major drawback of working with rhizotrons is the limited volume and hence limitations in time. This means that lupine plants grown for profit are likely grown longer than the 27 days of the experiment. For this reason, the nutrient and microbial dynamics observed in a longer-term experiment might be different than described here. As root architecture is fundamental for plant productivity, variations in root structure and length as a result of soil nutrient availability driven by plant–bacteria interactions were compared, showing also no differences between fertilizers. However, they were different in the root length and morphology compared with the no fertilized plants.

To the best of our knowledge, the presented study is the first study to use amplicon sequencing to assess the effect of struvite and organic fertilizer on the rhizosphere and the rhizosheath microbiome. We identified spp. belonging to *Rhodanobacter*, *Rhizomicrobium*, *Acidobacteria*, *Microbacterium*, *Chitinophaga*, *Actinomadura*, *Mucilaginibacter*, *Nocardioides*, *Burkholderia*, *Streptomyces* and TM7 *genus incertae sedis* as the bacterial genera with the highest relative abundance in the rhizosheath. Moreover, we showed that addition of struvite or an organic fertilizer to a growing medium influenced the microbial composition, in which oligotrophic microorganisms such as *Acidobacteria* decreased their relative abundance over time. These results suggest that the rhizosheath-associated bacterial community evolved into copiotrophic populations as a result of the increased N available in the growing medium. Our results confirm the high phenotypic diversity between representatives of the genus *Rhizobium* inhabiting the rhizosheath of lupine plants supplemented with different fertilizers. Moreover, analysis of the significant differences in taxa abundance uncovered a log2 fold increase in *Phenylobacterium* in growing medium supplemented with an organic fertilizer when compared with medium without fertilizer.

In contrast, this same genus was significantly more abundant in the growing medium supplemented with struvite. It is very well known from the literature that amplicon sequencing gives accurate information on microbial taxonomy (Poretsky et al., 2014), and in addition, different community metrics can be calculated, such as species richness, evenness, and diversity. However, this technique does not give data on the actual microbial biomass.

## Conclusion

The elucidation of plant–rhizosphere–soil interactions is necessary for understanding and improving fertilizer efficiency. Hence, the use of recovered products such as struvite and organic fertilizers needs to be accompanied by specific rhizosphere analyses to increase plant nutrient use efficiency and therefore, yields.

Based on our results, both N sources applied increased plant biomass to a similar level; however, the N dynamic in the growing medium showed significant differences between struvite and organic fertilizer treated plants. After N balances, it was shown that the N from the organic fertilizer was still in the form of organic N or was used by another system such as the native microbial community, or was lost to the atmosphere due to mineralization, hindering its quantification in the soil; however, most of the N released from struvite remained immobilized in the soil. Still, similar N mineralization was observed with organic and struvite treatments. That might explain the comparable root growth between struvite- and organic-treated lupine plants contrary to what was observed in the rhizosphere of other species such as tomato where the mineralization of ammonium from struvite did not occur. Consequently, higher root length was observed in the organic fertilizer vs. struvite treated plants. Also, even if the N recovery was higher with the organic fertilizer compared to struvite, no significant differences in biomass were observed.

Furthermore, the low N recovery (in both cases, less than 5%) was enough to show significant differences between the growth of fertilized and unfertilized plants. With lupine plants, the fertilizer applied seems to have a significant impact on the associations and functionality of the bacteria inhabiting the growing medium used, suggesting that fertilizer influenced the interindividual dissimilarities in the most abundant genera between treatments. This means that different species have a distinct effect on modulating the associated microbial community, but in the case of lupine, the fertilizer had a more significant effect than the plant itself.

It was shown that the products recovered can substitute the use of mineral fertilizers and therefore have a commercial value. The research, therefore, promotes the recycling of recovered products to a greater extent. Furthermore, the use of lupine as an economically relevant crop was of central interest. There is no doubt that generating a detailed understanding of rhizosheath–rhizosphere-related microbial community, their assembly over time and activity will be essential to manipulate root–soil interactions and to ensure sustainable fertilizer use efficiency and soilless crop production in the future.

## Data Availability Statement

The raw16S rRNA reads have been made available on the SRA under accession number ID PRJNA623265. Original R scripts are available in GitHub (https://github.com/ehdezsanabria/Lupine).

## Author Contributions

AR-A, OG, and NDJ conceived and designed the experiments. AR-A and OG performed the experiments. MM performed data analysis and revision of the manuscript. EH-S processed the Illumina libraries and performed the data mining, statistical analysis, interpretation, and figure and table preparation of all 16S rRNA amplicon sequencing results. OG and AR-A completed the statistical data processing of the physicochemical variables measured. NDJ and NB contributed to the revisions of the manuscript. AR-A, OG, EH-S, and NDJ wrote the manuscript. EM contributed to the discussion of data and revision of the manuscript. All authors contributed to the article and approved the submitted version.

## Conflict of Interest

The authors declare that the research was conducted in the absence of any commercial or financial relationships that could be construed as a potential conflict of interest.
