# Supplementary material for: Effect of Applying Struvite and Organic N as Recovered Fertilizers on the Rhizosphere Dynamics and Cultivation of Lupine (Lupinus angustifolius)
Source: Front Plant Sci. 2020 Nov 19;11:572741. doi: 10.3389/fpls.2020.572741 (PMC7717983; doi:10.3389/fpls.2020.572741)
Supplement: Supplementary file 1 [file Data_Sheet_1.docx]

**Supplementary Information**

**Effect of applying struvite and organic N as recovered fertilizers on the rhizosphere dynamics and cultivation of lupine (*Lupinus angustifolius*)**

**Ana A. Robles-Aguilar^1,2+^, Oliver Grunert ^3,4+^, Emma Hernandez-Sanabria^3,5^, Mohamed Mysara^6,7^,** **Erik Meers^1^, Nico Boon^3*^, Nicolai D. Jablonowski^2*^**

^1^Department of Green Chemistry and Technology, Faculty of Bioscience Engineering, Ghent University, Coupure Links 653, 9000 Gent, Belgium

^2^Forschungszentrum Jülich GmbH, Institute of Bio- and Geosciences, IBG-2: Plant Sciences, 52428 Jülich, Germany

^3^Center for Microbial Ecology and Technology (CMET), Ghent University, Coupure Links 653, 9000 Gent, Belgium

^4^Greenyard Horticulture, Skaldenstraat 7a, 9042 Gent, Belgium

^5^VIB – Center for Microbiology, KU Leuven Laboratory of Molecular Bacteriology, Rega Institute, Herestraat 49 - Bus 1028, 3000 Leuven, Belgium

^6^Unit of Microbiology, Belgian Nuclear Research Center (SCK•CEN), Mol, Belgium

^7^Department of Bioscience Engineering, Vrije Universiteit Brussel, Brussels, Belgium

+these authors contributed equally to this work.

***Correspondence:**

Dr. Nicolai D. Jablonowski, Institute of Bio- and Geosciences, IBG-2: Plant Sciences, Forschungszentrum Jülich GmbH, 52425 Jülich, Germany, Phone +49-2461-61-8682 Fax -+49-2461-61-2492, e-mail: n.d.jablonowski@fz-juelich.de

Nico Boon, Center for Microbial Ecology and Technology (CMET), Ghent University, Coupure Links 653, 9000 Gent, Belgium, Phone: +32 (0)9 264 59 76, e-mail: Nico.boon@ugent.be

**Supplementary Figure 1. Beta diversity of rhizosheath (A) and rhizosphere (B) bacterial communities in growing media with or without lupine plants.** Circles indicate communities harbouring lupine plants, and diamonds show growing medium without plants. Yellow indicates communities in growing medium that were supplemented with organic fertilizer, while blue indicates addition of struvite and orange is no fertilizer application. Samples of gamma-irradiated soil were followed over time to observe the bacterial community development in the rhizosphere (B). The growing medium harbouring plants was not sterile. PERMANOVA results indicate that time and pre-treatment of the growing medium (sterile or not sterile medium) are the factor contributing explaining the highest percentage of the variance among communities in the rhizosphere. On the contrary, time and fertilizer are equally significant for the dissimilarity in bacterial communities in the rhizosheath (A).


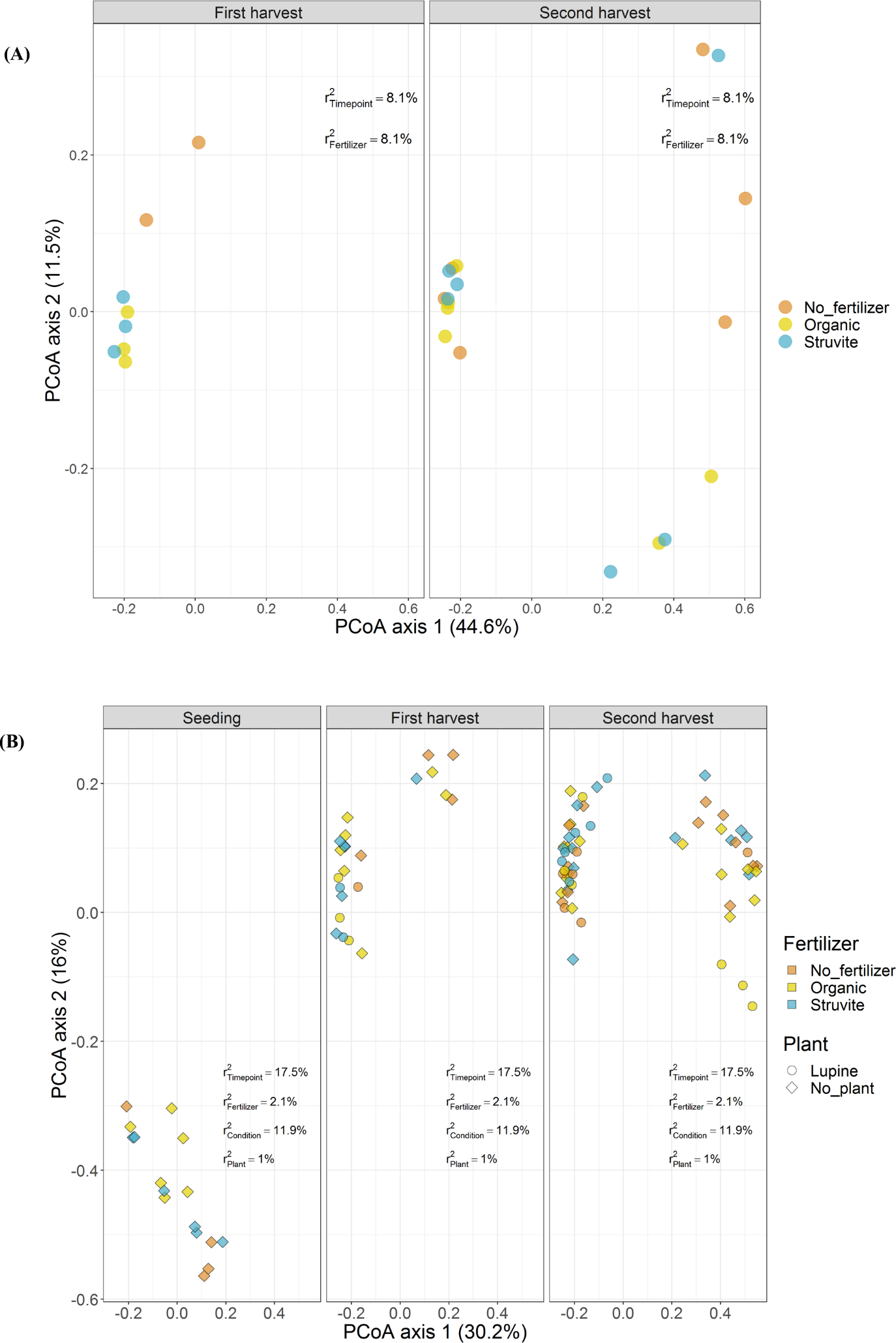


**Supplementary Figure 2. Fertilizer and plant presence were determinant for impacting community characteristics:** alpha diversity in the rhizosheath (A) in the rhizosphere of growing medium without plant (B, upper panel) and containing lupine (B, lower panel).observed diversity (richness) in rhizosheath (C) and rhizosphere (D), and evenness in rhizosheath (E) and rhizosphere (F).


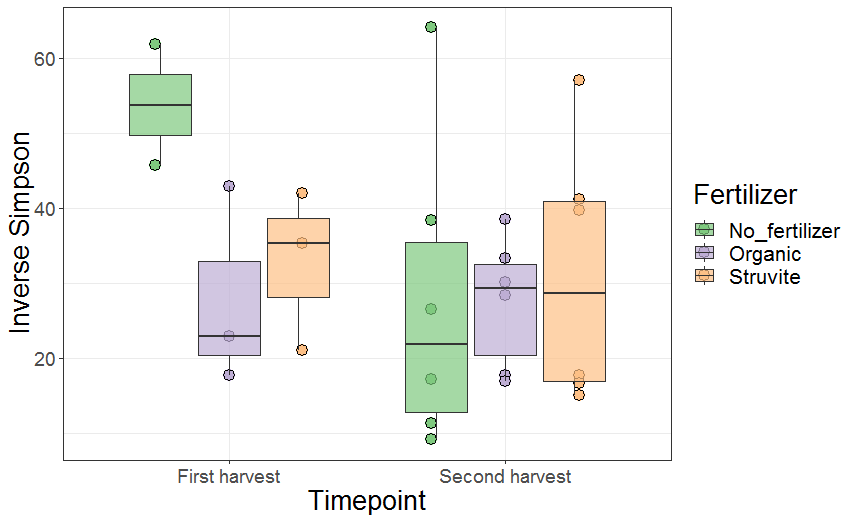


**(A)**


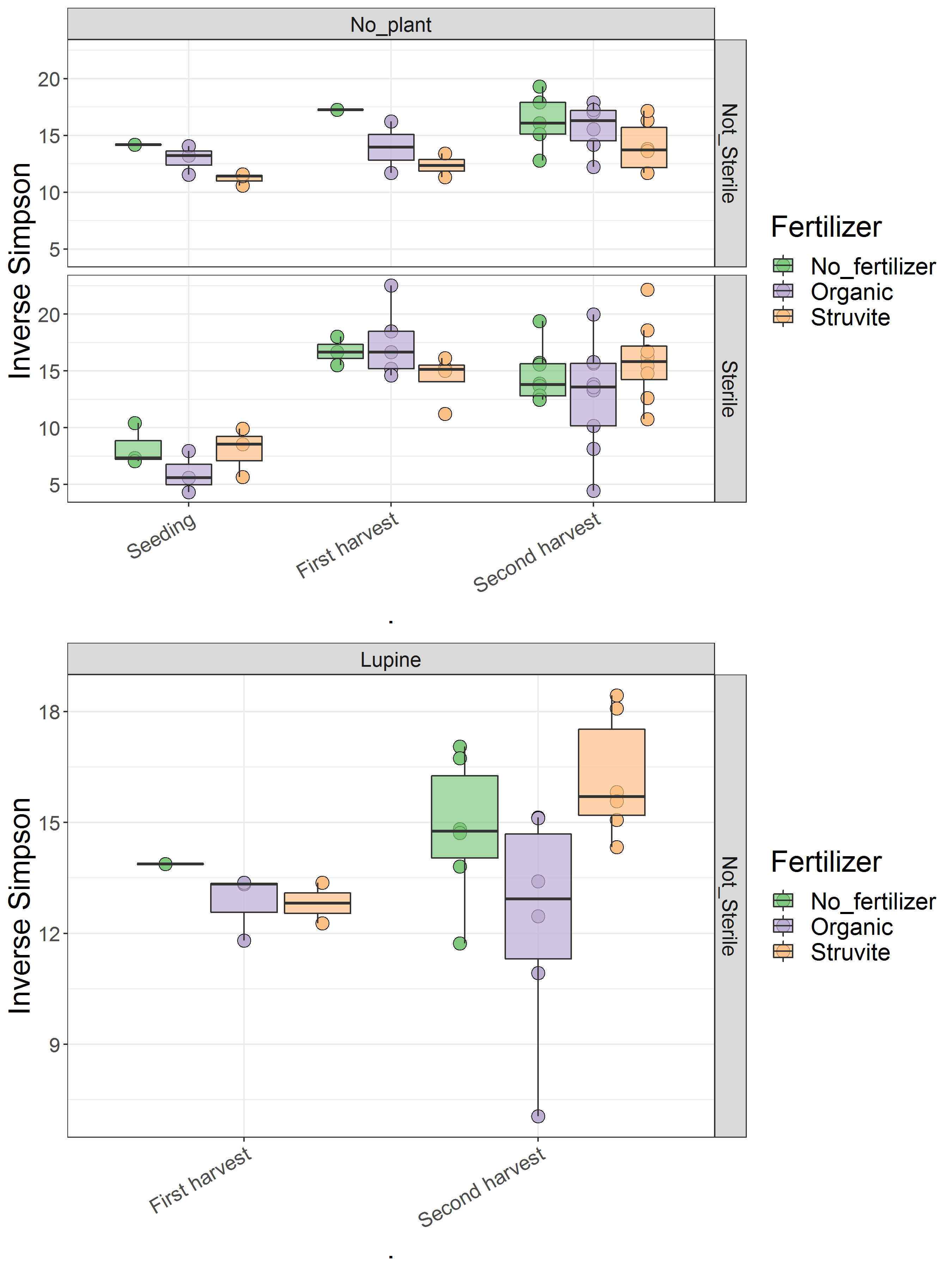


**(B)**


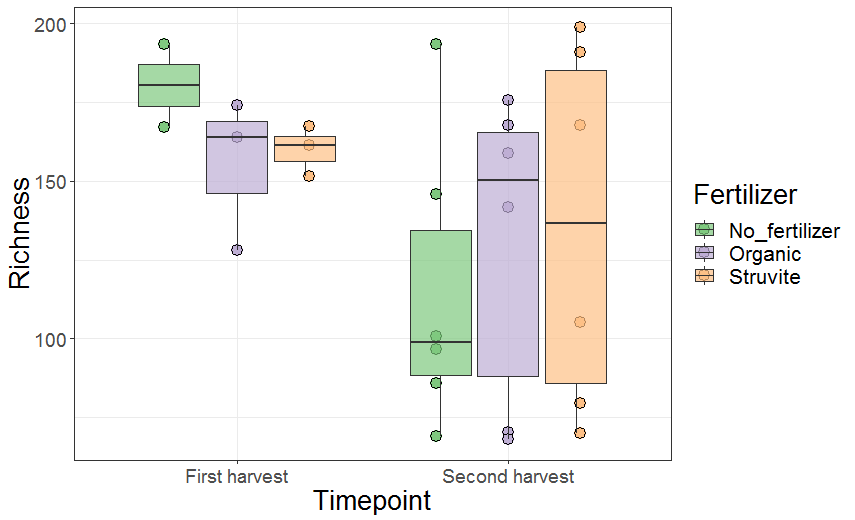


**(C)**


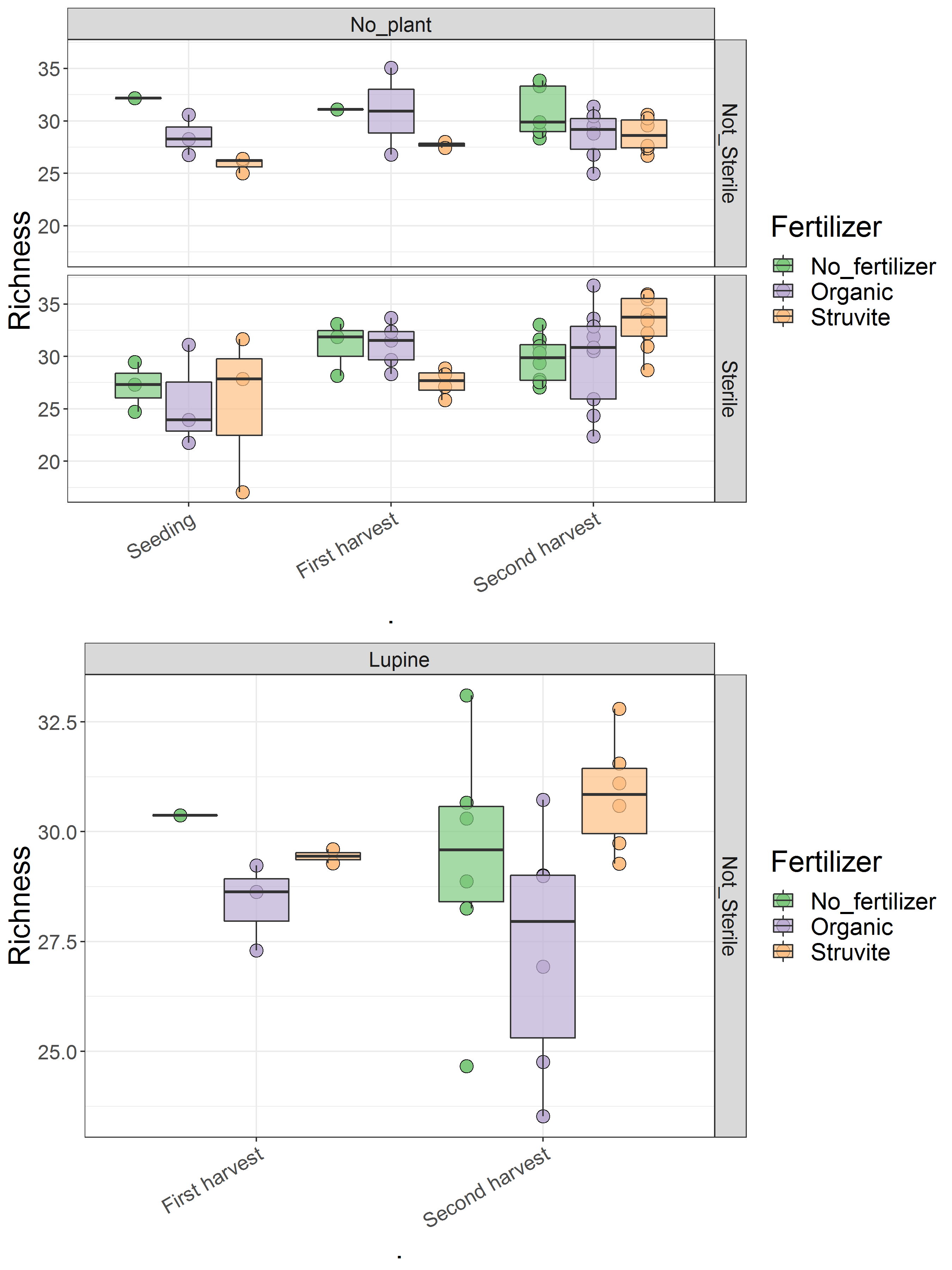


**(D)**


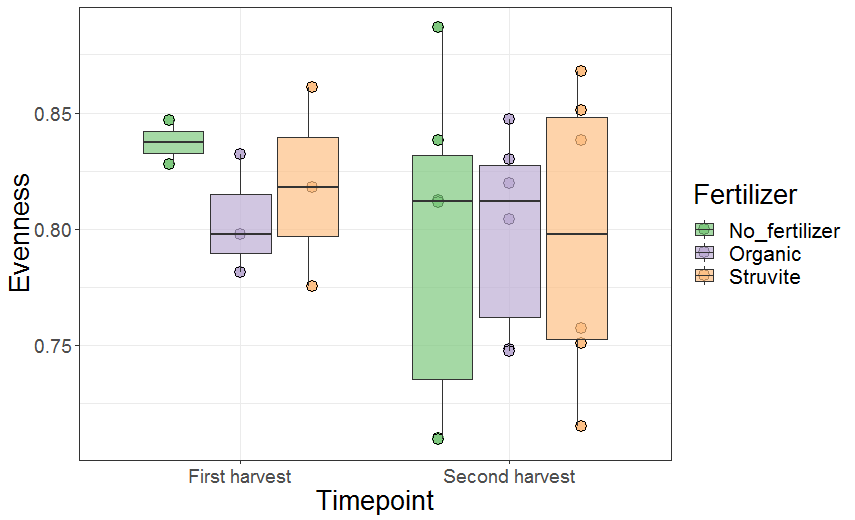


**(E)**


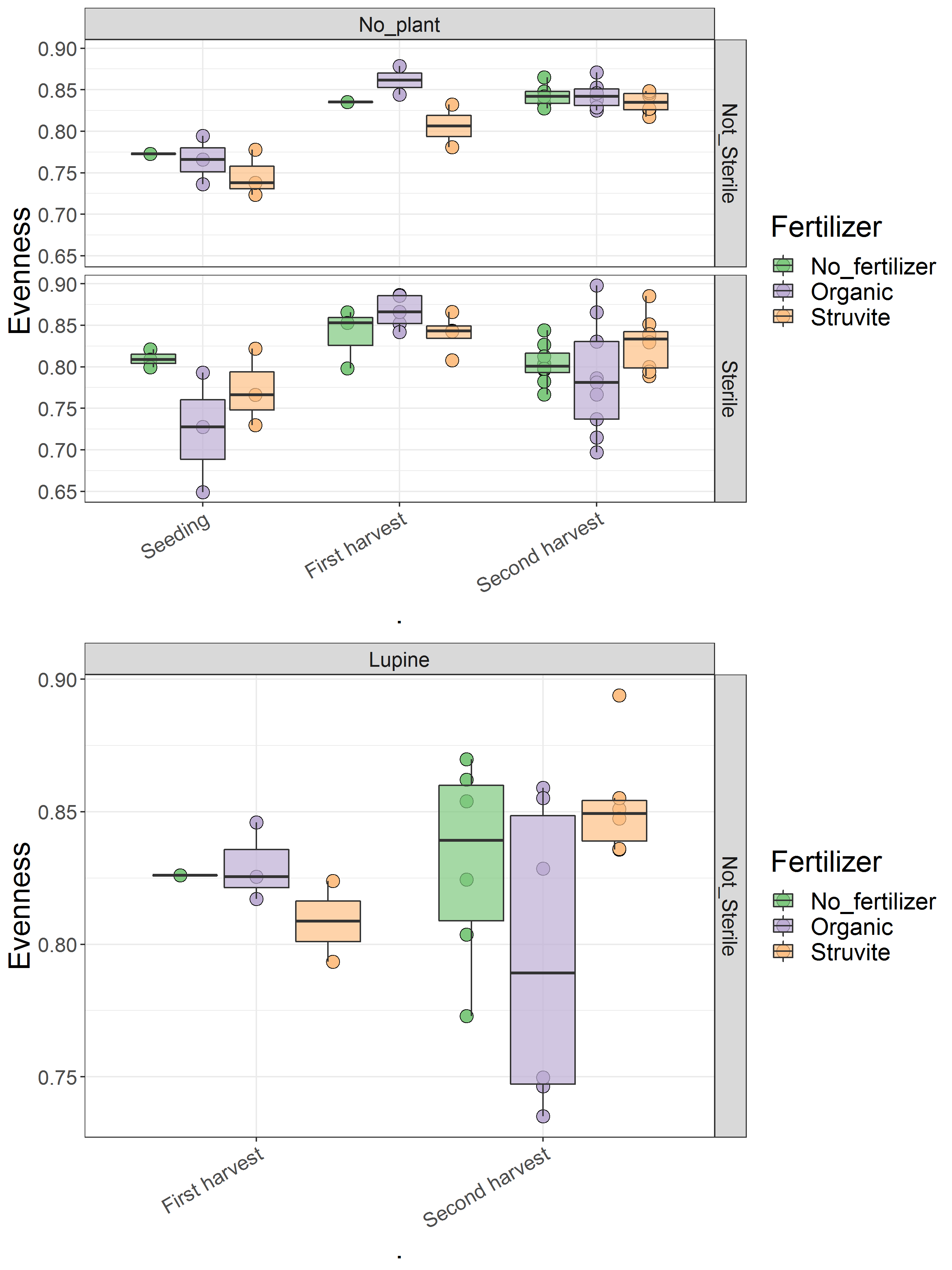


**(F)**

**Supplementary Table 1. Average cm of primary, secondary and tertiary root length per measurement day for each treatment.** A similar distribution of primary-thicker roots and secondary-thinner roots between organic and struvite fertilizers was observed. At tp1 (measurement day 4) primary root counted 15% of the total root length for the struvite treated plants, and for organic was 14%. Secondary root length was 84% in both treatments, and tertiary roots counted for 1% in struvite and 2% in organic fertilizer. Primary roots were up to 20% of the total root length when no fertilizer was applied.

|  | **Measurements days** | | | | | |
| --- | --- | --- | --- | --- | --- | --- |
|  | 1 | 2 | 3 | 4 (tp1) | 5 | 6 (tp2) |
| Length (cm) | **No fertilizer (NoF)** | | | | | |
| Primary root | 17.6±6 | 23.1±12 | 27.5±17 | 30.3±21 | 11.7±4 | 11.7±4 |
| Secondary root | 2.4±2 | 24.4±18 | 52.3±33 | 123.5±94 | 247.7±36 | 331.6±51 |
| Tertiary root | 0.0±0 | 0.0±0 | 0.2±0.7 | 0.7±1 | 4.8±3 | 63.8±24 |
| **Total** | 20.0±5 | 47.4±9 | 80.1±18 | 154.5±76 | 264.2±34 | 407.1±67 |
|  | **Organic (ORG)** | | | | | |
| Primary root | 12.0±6 | 15.7±12 | 18.2±16 | 19.5±17 | 7.3±0.6 | 7.3±0.6 |
| Secondary root | 5.1±4 | 31.0±22 | 62.9±31 | 115.0±63 | 195.8±30 | 260.3±49 |
| Tertiary root | 0.0±0 | 0.0±0 | 0.2±0.6 | 2.5±3.3 | 11.8±3 | 60.5±25 |
| **Total** | 17.1±4 | 46.6±12 | 81.3±17 | 137.0±51 | 214.9±31 | 328.1±69 |
|  | **Struvite (STR)** | | | | | |
| Primary root | 12.6±5 | 14.9±9 | 16.6±12 | 18.1±14 | 13.1±7 | 19.0±16 |
| Secondary root | 5.1±6 | 27.1±17 | 55.0±26 | 100.1±57 | 185.1±96 | 227.0±84 |
| Tertiary root | 0.0±0 | 0.0±0 | 0.0±0 | 1.1±3 | 15.3±16 | 40.6±32 |
| **Total** | 17.7±5 | 41.9±12 | 71.6±20 | 119.3±49 | 213.5±111 | 286.6±103 |

*Values of the total length indicated for each measurement day correspond with the values in the graph in Figure 2.
